# Insuppressible cognitions in the reflexive imagery task: Insights and future directions

**DOI:** 10.3389/fpsyg.2022.957359

**Published:** 2022-10-12

**Authors:** Jessica K. Yankulova, Lisa Moreno Zacher, Anthony G. Velasquez, Wei Dou, Ezequiel Morsella

**Affiliations:** ^1^Department of Neurosurgery, Stanford University School of Medicine, Stanford, CA, United States; ^2^Department of Psychology, San Francisco State University, San Francisco, CA, United States; ^3^Department of Psychology, University of California, Santa Cruz, Santa Cruz, CA, United States; ^4^Neuroscape, Department of Neurology, University of California, San Francisco, San Francisco, CA, United States

**Keywords:** reflexive imagery task, unconscious processing, consciousness, encapsulation, involuntary entry

## Abstract

In 1959, Neal Miller made the bold claim that the Stimulus–Response, Behaviorist models of that era were describing the way in which stimuli lead to the entry of contents into consciousness (“entry,” for short). Today, researchers have begun to investigate the link between external stimuli and involuntary entry, using paradigms such as the reflexive imagery task (RIT), the focus of our review. The RIT has revealed that stimuli can elicit insuppressible entry of high-level cognitions. Knowledge of the boundary conditions of the RIT effect illuminates the limitations of involuntary processes and the role of consciousness in the regulation of behavior. We review the boundary conditions of this paradigm as well as its systematic effects. Systematic effects are unlikely to be due to experimental demand. While reviewing each effect, we consider its theoretical implications. In addition, throughout our review, we discuss future directions for the study of insuppressible entry using the RIT. Last, we discuss a theoretical development (passive frame theory) that stems from the RIT and illuminates how involuntary entry and encapsulation, though at times disadvantageous for the actor, are essential for adaptive *action selection* during the course of ontogeny.

## Introduction

In 1959, during the end of the era of Behaviorism, Neal Miller, one of the leading experimentalists of that era, concluded something that went against the principal tenets of Behaviorism. He proposed that the Stimulus–Response models of that era were describing, not only the links between stimuli and overt behavior, but the manner in which stimuli lead to the entry of phenomenal contents into consciousness (“entry,” for short; Miller, 1959). Consciousness was then a taboo phenomenon, falling outside the domain of traditional Behaviorism. Today, the nature of entry continues to be a mystery (Jerath and Beveridge, 2018).

Consistent with Miller’s conclusion, after an unexpected nap on the beach, one might experience the smell of sunblock. In this way, to the actor, the activation of such a *conscious content* often “just happens” (Morsella et al., 2016). (Any particular thing one is conscious of has been referred to as a “conscious content.” The *conscious field* is made up of all the conscious contents that are activated at one moment in time.) Involuntary entry can also stem from a combination of external stimuli and the particular task set that is activated (*set-based entry*; Bhangal et al., 2018), which is different in nature from the entry of percepts and urges. (A set is the disposition to act or think in a certain manner.) Ach (1905/1951) observed that, if one activates the set to divide before hearing “two and two,” then one will think “one.” Had the activated set been to subtract, however, then one would think “zero.”

Researchers have begun to heed Miller’s conclusion and begun to investigate the link between external stimuli and insuppressible entry, using variants of the Stroop task (Stroop, 1935; Morsella et al., 2009a), the flanker task (Eriksen and Eriksen, 1974; Morsella et al., 2009b), and the reflexive imagery task (RIT; Allen et al., 2013), the focus of this article. Below, we review some RIT effects and consider their theoretical implications. In addition, throughout our review, we discuss future directions for the study of insuppressible entry using the RIT.

### The reflexive imagery task

**Figure 1:**
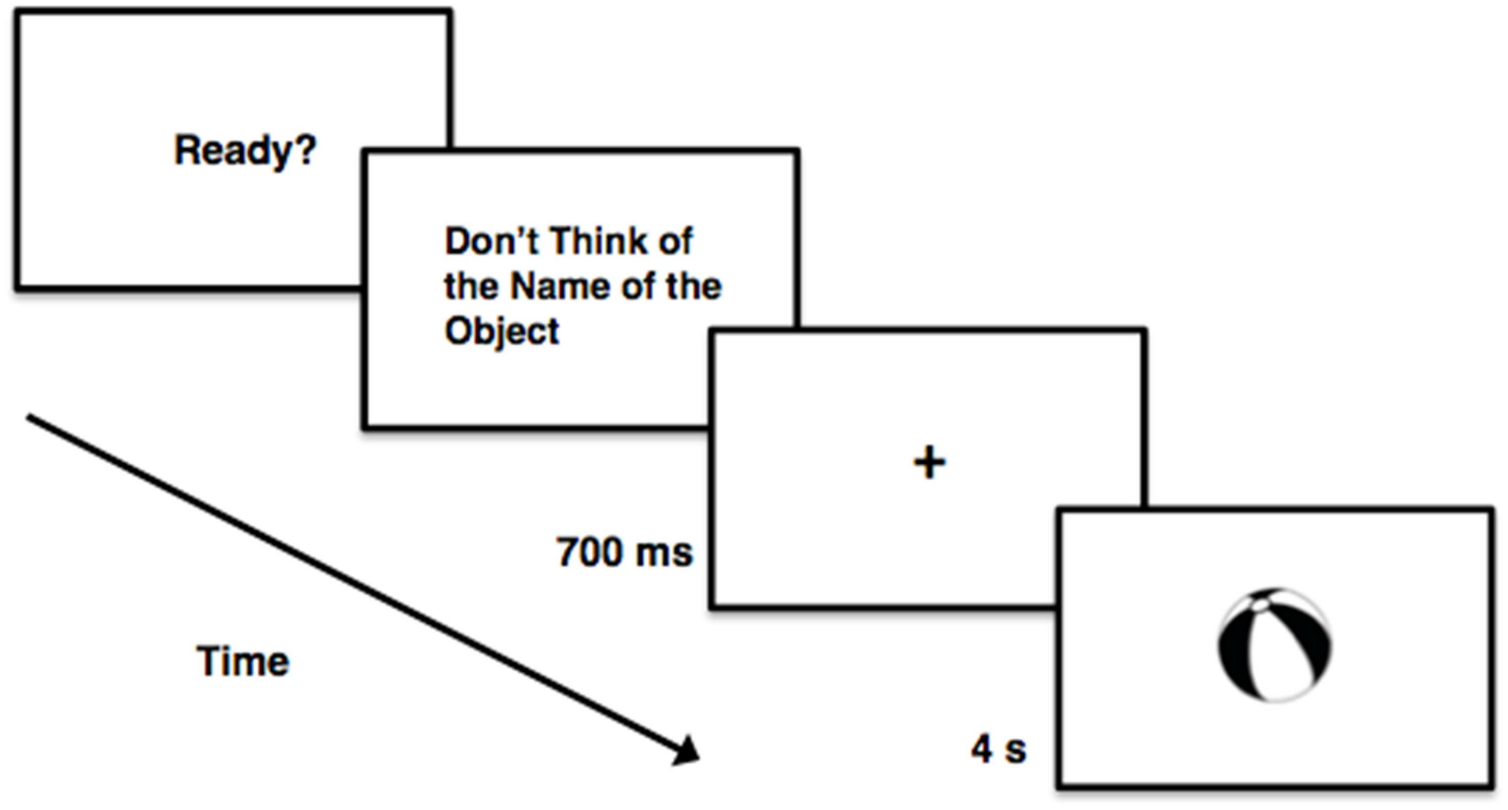
Schematic depiction of an RIT trial (not drawn to scale).

The RIT was developed to investigate set-based entry. The RIT stems from variants of the Eriksen flanker task (e.g., Morsella et al., 2009a,b), theoretical developments (Morsella et al., 2016), and experimental findings (Ach, 1905/1951; Uznadze, 1966; Eriksen and Eriksen, 1974; Wegner, 1989; Gollwitzer, 1999; Morsella et al., 2016). In the task, participants are instructed not to perform a given mental operation in the presence of certain stimuli. For example, participants might be instructed not to subvocalize the name of a to-be-presented stimulus (Allen et al., 2013; Figure 1).^1^ To subvocalize is to name in one’s mind but not aloud. On a substantive proportion of the trials (Allen et al., 2013), the RIT effect arises: the stimulus CAT yields the activation of “cat” (i.e., /k/, /œ/, and /t/).

RIT effects can rely on operations more complex than that of the subvocalization of the names of objects. In Merrick et al. (2015), for example, participants were presented with visual objects and instructed not to perform two tasks: think of the name of the object, and count the number of letters in the object name. Both mental operations occurred involuntarily on ~30% of the trials. In another, complex version of the task, Cho et al. (2016) employed the childhood game of Pig Latin. In this study, participants first learned to transform words according to the game. Afterward, participants were presented with words and instructed not to transform them. Insuppressible transformations arose on a substantive proportion of trials (~ 40%). It is worth noting that this effect requires symbol manipulation, which is an operation associated with the frontal cortex (Miller and Cummings, 2007). For discussion of the neural correlates of the basic RIT effect, see Dou et al. (2020).

In addition, RIT effects have arisen from (a) syntactic processing (Bui et al., 2019); (b) mental rotation (Cushing et al., 2019); (c) musical imagery (White et al., 2018); (d) high-level shifts in spatial attention (Gardner et al., 2020); (e) insight-related processes (e.g., the insight that “candle” is associated with the stimuli WAX and FLAME; Bui et al., 2019); (f) and the type of sophisticated visuospatial imagery that occurs in chess (Cushing et al., 2019).

### Systematic effects and the validity of participants’ self-reports

Inaccurate self-reports in an RIT could arise from errors in memory (Block, 2007; see discussion in Morsella et al., 2009b), confabulations, or demand characteristics. Corroboration of participants’ self-reports stems from several sources, including systematic effects. (Systematic effects are experimental effects that are orderly, organized, and, because of their methodically arranged nature, are unlikely to be spurious.) First, when participants performing an RIT (Cushing et al., 2017) were instructed to report both the occurrence of the basic RIT effect and whether the subvocalization rhymed with a word held in mind, accuracy was high (> 80%). Such an effect could arise only if the participant retrieves the phonological form of a word. Second, in another RIT (Bhangal et al., 2018), participants were presented with a set of visual objects (e.g., three dots) and instructed not to count the stimuli. Accuracy of the insuppressible counting was high (~90% mean accuracy) for the condition in which the number of objects was small. This finding suggests that the counting did in fact occur, which is in line with participants’ introspections. Third, the effects in RITs involving insuppressible subvocalizations are influenced by word frequency such that high-frequency words are more likely to yield an effect than low-frequency words (Bhangal et al., 2015). This is a systematic effect, one that is unlikely to arise from demand characteristics. Fourth, the effect often arises too quickly to be caused by strategic processing (Allen et al., 2013; Cho et al., 2014). In Bhangal et al. (2015), participants reported on a substantive proportion of the trials (mean proportion = 0.71, *SE* = 0.03) that the subvocalization effect felt “immediate.” Fifth, RIT effects are more likely to arise for some sensory systems than for others. For example, RIT effects are more likely for verbal and visual imagery than for olfactory/gustatory imagery (Dou et al., 2018). Such a systematic effect is unlikely to arise from demand characteristics. Last, the RIT effect still arises when there is cognitive load, a condition in which it is difficult for participants to implement any form of strategic processing (Cho et al., 2014).

In Cho et al. (2014), participants attempted to thwart the RIT effect by reiteratively subvocalizing a speech sound (“da, da, da”) while the visual stimulus object was present. The RIT effect still arose in over 80% of the trials. Perhaps the RIT effect arose only because of the pauses, which were moments of silence, between the speech sounds. However, this hypothesis is inconsistent with the observation that the same results were obtained when participants subvocalized a continuous hum (“daaa….”). Perhaps the RIT effect might have been thwarted if the phonological store had been occupied with verbal information of a more complex nature (e.g., words, as in poem). This possibility could be evaluated in future research.

There are other cases in which manipulations of cognitive load do not seem to decrease the likelihood of an RIT effect. For example, Walker (2019) found substantive rates of insuppressible subvocalizations (~50% of the 30 trials) even when the stimulus was presented briefly (< 200 ms) or when the stimulus was presented under conditions of perceptual load (the stimulus was encircled by six other line drawings). In another experiment (Velasquez et al., 2021), stimulus-elicited insuppressible imagery was observed even when the visual, eliciting stimuli were presented within a complex, dynamic scene (a movie) and the participant was engaged in secondary tasks that are known to induce cognitive load.

### The insuppressible nature of the RIT effect

There is a theoretical basis for the claim that the RIT effect is involuntary. Wegner (1994) proposes that ironic effects, including the RIT effect, stem from a “monitoring” process that is automatic. To other theorists (Ach, 1905/1951; Bhangal et al., 2016), these effects are the result of sets being automatically activated by instructions. From this point of view, just by hearing the word “add” in the instruction “Do not add the following numbers,” there is activation of the set to perform this mental operation. This notion is consistent with the principles of *parallel distributed processing* (Rumelhart et al., 1986).

Last, theorists have posited that RIT effects stem from the “encapsulated” nature of the production of the majority of conscious contents (Fodor, 1983; Morsella et al., 2016). Perceptual illusions are said to be encapsulated, because knowledge of the true nature of the perceptual stimuli cannot affect the illusion. The notion of encapsulation (discussed below) is consistent with the aforementioned idea that one is aware of the outputs of mental operations but not of the operations themselves (Helmholtz, 1856/1925; Lashley, 1956; Miller, 1959, 1962; Nisbett and Wilson, 1977).

In summary, in all theoretical accounts of the RIT effect, the effect is involuntary.

### Boundary conditions of the RIT effect

The RIT provides a technique that can test the limits of involuntary processes without relying on subliminal stimuli.^2^ Subliminal stimuli can be problematic because these imperceptible stimuli are not only unconscious, but they are also of weak strength (Bargh and Morsella, 2008). Most unconscious processes operate over stimuli of greater strength. The RIT could be construed as involving the Helmholtzian-Freudian unconscious, an unconscious that operates over supraliminal stimuli.

Knowledge of the boundary conditions of these insuppressible effects could shed light on the limitations of involuntary processes and on the role of conscious processes in the control of thought and behavior. The effect will not arise for subliminal stimuli (see Acknowledgment): no RIT effects were observed with orthographs rendered subliminal through masking. In addition, RIT effects will also not arise for operations associated with autonomic functions (Bhangal et al., 2016). This observation supports the hypothesis that the RIT effect is associated with the corticospinal tract (Morsella et al., 2016). The effect will also not arise for processes involving emotional/incentive systems. It is obvious that, regarding the control of emotion, one cannot by sheer will and without some difficulty make oneself frightened or ecstatic. In addition, RIT effects will not arise for overt action: participants are capable of not uttering aloud the name of objects when instructed not to do so (Allen et al., 2013). Often, what is experienced as a very strong urge, or even as the strongest urge, is not what guides action selection and overt behavior (Morsella, 2005).

## Involuntary entry from the standpoint of passive frame theory

Findings from the RIT support the aforementioned conclusion that, in cognition, one is often aware only of the output of mental operations (Helmholtz, 1856/1925; Lashley, 1956; Miller, 1959, 1962; Nisbett and Wilson, 1977). Often, stimuli activate conscious contents in a direct, insuppressible manner. This is consistent with Miller’s (1959) bold proposal that the S-R models of that era were describing the reflex-like manner in which stimuli lead to the entry of contents into consciousness (Miller, 1959). According to Morsella et al. (2016), the insuppressible nature of the stimulus-elicited effects in the RIT, and the general arrangement in which such conscious contents cannot be suppressed, is advantageous during ontogeny (Morsella et al., 2016). Consider, for example, that the ability to deactivate voluntarily contents such as pain, nausea, or guilt would be detrimental: These contents serve a critical role in guiding behavior (Baumeister et al., 2007), especially during early development. Thus, it has been proposed that the encapsulation of conscious contents, though at times disadvantageous to the actor, is adaptive.

### Encapsulation

To understand the concept of encapsulation, it is useful to consider the famous Müller-Lyer illusion. The viewer experiencing the illusion is aware, in some sense, that the two horizontal lines are identical in length. Yet, the lines do not seem that way. Hence, the illusion is said to be protected or “encapsulated” (Firestone and Scholl, 2016; Pylyshyn, 1984) from the influence of the viewer’s knowledge about the stimulus. The conscious contents composing the field cannot influence each other and, figuratively speaking, are unaware of each other and of whether they are action-relevant (Morsella et al., 2016). These contents function as a lighthouse does: The lighthouse, always in operation, is unaware of which ships can see its light. From this standpoint, visual perception is modular and encapsulated from the rest of cognition, such that perception is “cognitively impenetrable” (Pylyshyn, 1984). Thus, what we perceive is “functionally independent from what and how we think, know, desire, act, and so forth” (Firestone and Scholl, 2016, p. 3).

It has been proposed that conscious contents might be encapsulated from *the will* of “the observer” as a result of the multidimensional, spatial structure of the conscious field, a structure in which, according to the rules of projective geometry, the observer must (a) be separated from all conscious contents and (b) not itself be a conscious content (Rudrauf et al., 2017; Merker et al., 2022). This is consistent with the view that the observer cannot directly apprehend, nor introspect about, itself (Schopenhauer, 1818/1819). According to Morsella et al. (2016), each content is encapsulated from the will of the observer and from the influence of the other contents composing the field at that time.

### Principles of operation: From encapsulated outputs to adaptive behavior

With the foregoing in mind, the question arises, “How does adaptive behavior arise from such an arrangement?” According to Passive Frame Theory (PFT; Morsella et al., 2016), which stems from the RIT, encapsulated contents can influence overt behavior collectively, but only through the conscious field. Outside the conscious field, the contents can influence behavior, but not collectively. Such unconscious behavior yields ‘un-integrated’ actions (Morsella and Bargh, 2011). The un-integrated actions can be sophisticated (e.g., the handling of tools in anarchic hand syndrome or in utilization behavior; Marchetti and Della Sala, 1998; Suzuki et al., 2012; Yamadori, 1997), but they are not guided by all the types of contextual information by which they should be guided. These actions lack the kind of context-sensitivity that is apparent in adaptive behavior (e.g., holding one’s breath while underwater).

### Conscious contents as action options

Conscious contents that do not influence overt behavior directly have been construed as ‘action options’ (Morsella et al., 2016). The notion of conscious contents as action options is consistent with the view that, for adaptive action, it is best for urges and other conscious contents to function, not as ‘programs’ that inflexibly and directly trigger action, but rather as ‘advice’ for possible actions (Agre and Chapman, 1990; Morsella et al., 2016). It would be detrimental for stimulus-specific action plans, though activated to some extent by external stimuli, to directly control action selection and influence overt behavior. Action selection is adaptive when it takes into account all the current activations, from the stimulus scene, drives, memory, etc. These conscious contents, including the manner in which the spatial locations of these contents are represented (both with respect to each other and to the actor), are essential for adaptive action selection. It is for this reason that adaptive action occurs in a manner that is context-sensitive, on the fly, and flexible. Interestingly, motor programming, too, displays these qualities. A largely unconscious process, motor programming is computed “online” in a dynamic and context-sensitive manner (Rosenbaum, 2002).

Action plans used in the past might not be suitable for the current context. As General Eisenhower noted, “Plans are worthless, but planning is everything.” According to PFT, action selection must occur in the frame of all the other conscious contents activated at that one instant. This is called a ‘frame check’ (Morsella et al., 2016). For a successful frame check, the conscious field must operate quickly and be thorough regarding what it represents. And it does: Just as a loudspeaker can reproduce, and simultaneously present, the many sound waves (frequencies) produced by multiple musical instruments (all through the vibrations of a single diaphragm), the conscious field can present, somehow and with great speed and accuracy, a wide variety of conscious contents at one moment in time. From the present standpoint, consciousness is associated with a stage of processing that involves action options.

### The circumscribed role of the conscious field

**Figure 2:**
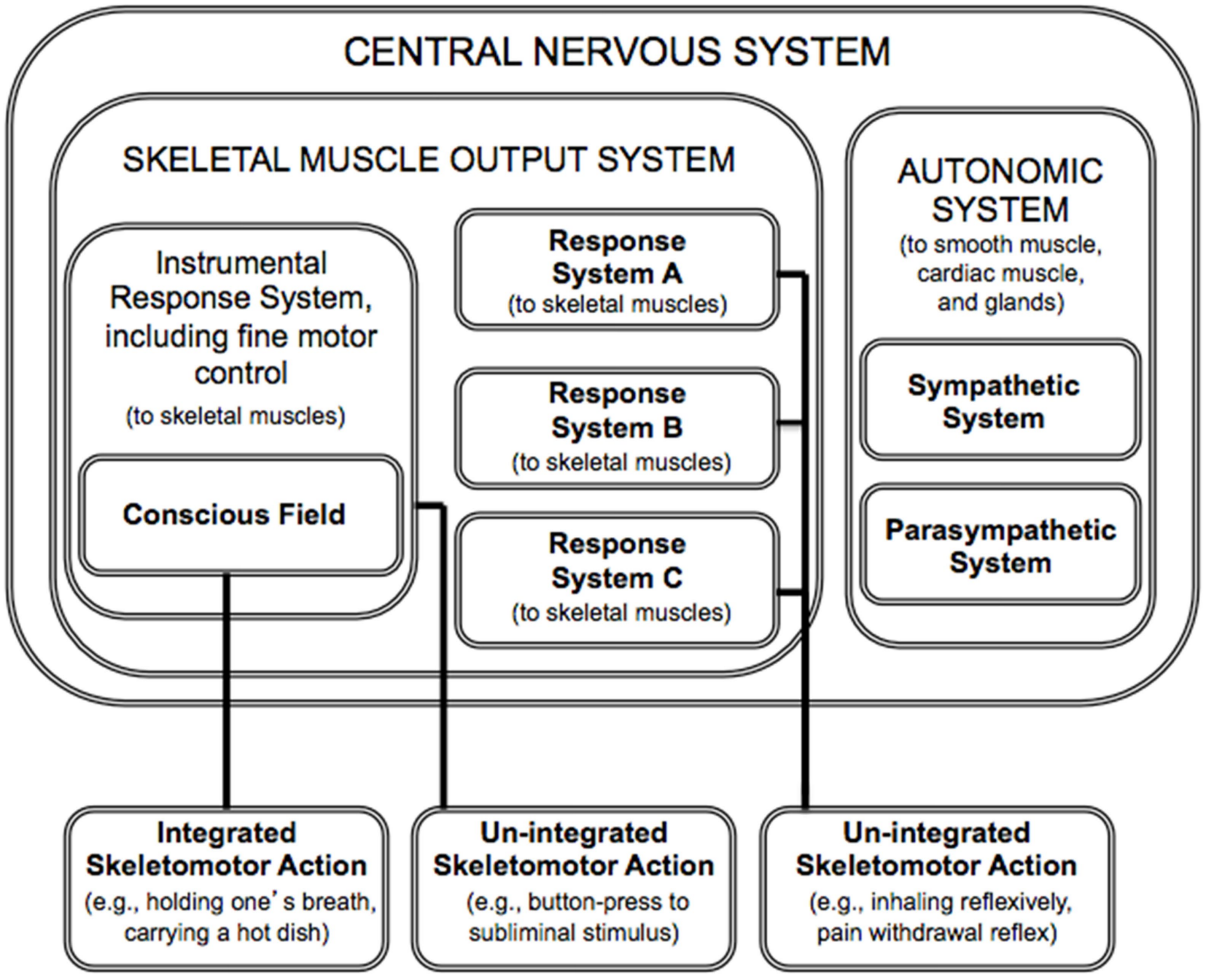
The circumscribed domain of consciousness within the nervous system (based on Poehlman et al., 2012; Morsella et al., 2016). Response systems can influence action directly, as in the case of “un-integrated” actions. It is only through the conscious field that multiple response systems can influence action collectively, as when one holds one’s breath while underwater (a case of “integrated” action).

The conscious field is sampled only by the *skeletomotor output system*, which is in the service of the somatic nervous system (Figure 2). The somatic nervous system is often contrasted with the autonomic nervous system. [It is an interesting question, for future research and theorizing, whether a single conscious field is servicing the multiple responses systems (of the skeletomotor output system; Morsella et al., 2016) or whether each effector system possesses its own conscious field; see relevant finding in Rapp et al., 2015.]

PFT reveals how the kind of reflexive mechanism proposed by Miller (1959) can, when part of a system composed of many such reflexive mechanisms, yield actions that are context-sensitive and more sophisticated than actions from a reflex arc, which, through stimulus-control, directly control overt behavior (e.g., the pupillary and patellar reflexes). The RIT findings reported here illuminate the nature of these reflexive mechanisms whose outputs populate and compose the conscious field. These theoretical developments would be of interest to researchers from disparate fields of study, including perception-and-action, cognitive control, and psychopathology.

## Author contributions

All authors listed have made a substantial, direct, and intellectual contribution to the work and approved it for publication.

## Conflict of interest

The authors declare that the research was conducted in the absence of any commercial or financial relationships that could be construed as a potential conflict of interest.

## Publisher’s note

All claims expressed in this article are solely those of the authors and do not necessarily represent those of their affiliated organizations, or those of the publisher, the editors and the reviewers. Any product that may be evaluated in this article, or claim that may be made by its manufacturer, is not guaranteed or endorsed by the publisher.

## Footnotes

^1^RIT effects arise in versions of the task that lack negative instructions (e.g., see the Baseline Condition in Allen et al., 2013). To take one example, Allen et al. (2016) instructed participants to hold in mind, as long as possible, one way of perceiving an ambiguous object (e.g., the Necker cube). Involuntary "perceptual reversals," which involved involuntary entry into consciousness, occurred on around 80% of the trials.

^2^The paradigm can also be used with special populations, such as those suffering from aphasia, in which, though overt naming is impaired, subvocalizing can be spared. It can also be used to investigate psychopathological phenomena in which intrusive cognitions play a role (e.g., ruminations and obsessions; see Magee et al., 2012).
